# Signal Processing for Novel Noise Radar Based on de-chirp and Delay Matching

**DOI:** 10.3390/s24227169

**Published:** 2024-11-08

**Authors:** Xinquan Cao, Shiyuan Zhang, Ke Tan, Jianchao Yang, Xingyu Lu, Zheng Dai, Hong Gu

**Affiliations:** School of Electronic and Optical Engineering, Nanjing University of Science and Technology, Nanjing 210094, China; 216104000029@njust.edu.cn (X.C.); zsy1997@njust.edu.cn (S.Z.); tank@njust.edu.cn (K.T.); ee_luxingyu@njust.edu.cn (X.L.); daizheng@njust.edu.cn (Z.D.); guhong666@126.com (H.G.)

**Keywords:** noise frequency modulation, linear frequency modulation, de-chirp, reduced sampling rate, delay matching

## Abstract

Modern radar technology requires high-quality signals and detection performance. However, traditional frequency-modulated continuous wave (FMCW) radar often has poor anti-jamming capabilities, and the high sampling rates associated with large time-bandwidth product signals can lead to increased system hardware costs and reduced data processing efficiency. This paper constructed a composite radar waveform based on noise frequency modulation (NFM) and linear frequency modulation (LFM) signals, enhancing the signal’s complexity and anti-jamming capability. Furthermore, a method for optimizing the processing of echo signals based on de-chirp and delay matching is proposed. The locally generated LFM signal is used to de-chirp the received echoes, resulting in a narrowband difference frequency noise signal. Subsequently, delay matching is performed in the fast time domain using the locally generated NFM signal according to the number of sampling points in the traversal processing period, allowing for the acquisition of target delay information. While reducing the analog-to-digital (A/D) sampling rate, the detection performance for wideband echo signals under high sampling rates is still maintained, with sidelobe levels and range resolution preserved. Accumulating this information in the slow time domain enables accurate target detection. The effectiveness of the proposed method is validated through simulation experiments.

## 1. Introduction

With the rapid advancement of modern radar technology, the importance of radar in medical, aviation, electronic warfare and other fields is becoming increasingly evident [1,2,3]. As a result, to meet the increasing demands, there is a pressing need to further enhance radar detection capabilities [4]. Improving signal quality and increasing the anti-jamming ability of detection signals are critical issues in radar applications [5,6]. Correspondingly, broadband signals have been widely applied in fields such as biology, military, and satellite navigation in recent years [7,8,9]. These signals, particularly when used as radar detection signals, offer numerous advantages, but they also pose certain challenges and issues.

From the perspective of signal reception, traditional frequency-modulated continuous-wave (FMCW) radar systems typically employ signals with a large time–bandwidth product to achieve long-range detection with high resolution. Linear frequency modulation (LFM) signals are the most commonly used transmission signals for this purpose [10]. LFM signals are relatively easy to generate, and the corresponding signal processing is also quite convenient. However, using high bandwidth imposes higher demands on the system’s sampling rate, making data acquisition significantly more challenging and leading to a substantial increase in hardware costs [11]. Moreover, even with hardware that meets the required conditions, the amount of data that the FMCW radar system needs to process remains substantial. This imposes significant pressure on system capacity and signal processing efficiency. On the other hand, as the electromagnetic environment in modern electronic warfare becomes increasingly complex and jamming technologies continue to advance, the detection performance of traditional LFM radars is increasingly constrained by various factors such as environmental interference and hardware limitations. Additionally, the simple nature of LFM signals makes them easily intercepted and identified by other platforms [12,13]. As a result, noise-based FMCW radar systems have gained widespread application in recent years. Noise frequency modulation (NFM) signals offer advantages such as low probability of interception, strong anti-jamming capabilities, and high electromagnetic compatibility [14,15,16]. However, due to the complex randomness of noise signals, their correlation is generally weak. Refs. [17,18] present an advanced pulse compression noise (APCN) waveform by combining LFM signals with noise signals, which effectively addresses certain issues and successfully implements imaging functions for synthetic aperture radar (SAR), while significantly reducing the probability of interception. Additionally, to achieve better detection performance, noise-based radars require the bandwidth of the transmitted signals to be increased as much as possible, which similarly leads to the challenge of needing a high sampling rate. In recent years, addressing the echo processing issues in detection [19,20,21], the traditional matched filtering method has been combined with fast orthogonal search [22,23]. This transforms the spectrum output of the matched filter into a line spectrum detection problem, enabling the accurate detection of target echoes and delay estimation. Additionally, for complex modulated radar signals, compressive sensing reconstruction technology has been integrated with matched filtering to obtain more precise target information [24,25]. However, these methods process the entire transmitted signal as a whole and do not address the need to reduce the sampling rate. Therefore, radar systems can consider combining NFM signals to increase the complexity and randomness of the transmitted signal. Subsequently, corresponding echo signal processing methods such as de-chirping and matched filtering can be applied to reduce the sampling rate while ensuring target detection performance.

This paper constructed a novel composite transmission waveform that combines NFM signals with LFM signals based on the operational context of an LFM radar system. Additionally, a signal optimization processing method based on de-chirp and delay matching is proposed, achieving reduced sampling rates and target detection. First, our radar system generates a wideband LFM signal and a narrowband NFM signal locally. These are modulated to form a novel composite transmit waveform that combines NFM with LFM. The signal exhibits a certain degree of randomness in both the time and frequency domains, offering strong anti-interception and anti-jamming capabilities. After the radar system receives the target echo, de-chirp is performed using the local LFM signal to obtain a narrowband NFM signal with a different frequency, thereby reducing the required sampling rate of the system [26]. The de-chirp echo is further matched with the different time-delayed versions of the locally generated NFM signal. The value of the corresponding delay sampling point index obtained from each match is used as the index value of the matching result. By traversing all the sampling points of the processing cycle, a result similar to matched filtering is obtained to acquire the target’s range information [27,28]. Finally, target detection is achieved through slow moving target detection (MTD) accumulation [29], which provides information on the target’s range and velocity. The feasibility of the proposed method is validated through simulation experiments.

The contributions of this article can be outlined as follows: (1) Constructing a novel composite radar transmission waveform that possesses anti-jamming capabilities and is difficult to detect. (2) Proposing a signal optimization processing method based on de-chirp and delay matching, which reduces the system sampling rate while maintaining detection performance. (3) The effectiveness of the proposed method is validated through simulation experiments.

This paper is organized as follows. Section 2 provides an overview of the research method’s background. Section 3 constructs the novel composite transmission signal model, which combines the NFM signal with the LFM signal. Section 4 proposes a signal optimization processing method based on de-chirp and delay matching. Section 5 demonstrates the effectiveness of the proposed method through simulation analysis. Finally, Section 6 summarizes this paper and discusses its limitations.

## 2. Background Description

**Figure 1:**
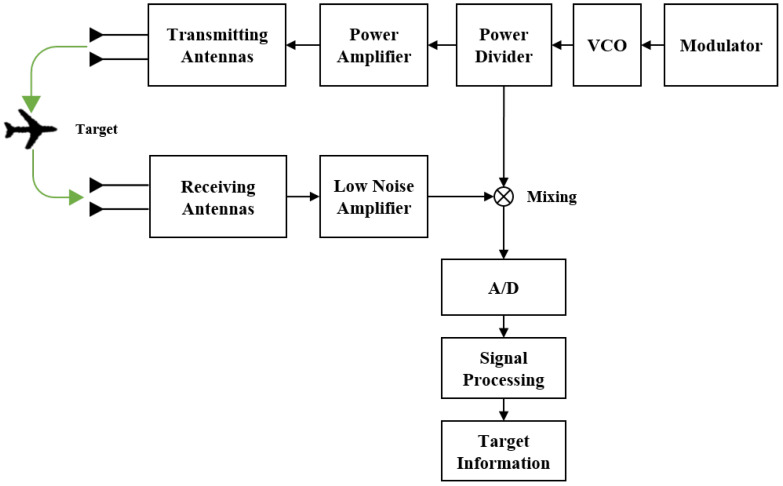
The structure of the conventional LFM continuous wave radar system.

This paper is based on the most typical traditional form of FMCW radar, using LFM signals for detection. The basic structure of a common LFM continuous wave radar system is illustrated in Figure 1. It primarily consists of components such as transceiver antennas, a power amplifier, power divider, voltage-controlled oscillator (VCO), modulator, analog-to-digital (A/D) converter module, and a signal processing module [30,31].

During the operation of the radar system, the modulator forms the signal generation module, which generates the modulation signal and then controls the VCO to produce the wideband LFM transmission signal. This signal is then passed through the power divider and transmitted as electromagnetic waves via the transmitting antenna. Simultaneously, a portion of the signal passing through the power divider is used as the local oscillator signal for the mixer. The electromagnetic waves transmitted by the antenna propagate through the atmosphere, scatter off moving targets, and form echo electromagnetic waves, which are received by the receiving antenna. The receiver processes these echoes through low-noise amplification, mixing, and filtering, after which they are converted into digital signals by the A/D converter. Finally, the signal processing module processes the signal to extract the target information.

However, because wideband LFM signals require the A/D module to operate at a high sampling rate, stable implementation can be challenging and costly. Therefore, this paper constructs a complex signal with anti-interference capability and the proposed method utilizes narrowband NFM signals for signal processing to reduce the sampling rate.

## 3. Signal Model

**Figure 2:**
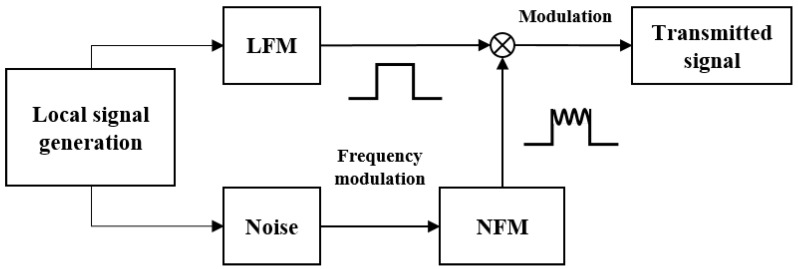
The structure of novel noise radar transmitted signal generation.

To enhance the complexity and randomness of the signal, this paper proposes a novel radar signal waveform constructed based on noise. The specific block diagram for generating the novel radar system’s transmitted signal is shown in Figure 2. Initially, a wideband LFM signal and a random noise signal are generated locally. A narrowband NFM signal is then produced based on the random noise signal. The local radar system combines the narrowband NFM signal with the wideband LFM signal through amplitude modulation, forming a composite signal. This composite signal is used as the transmitted signal for the radar system.

In the detailed description, the radar system generates a wideband LFM signal. Let the chirp rate of the signal be *K*, the time duration be $T_{r}$, the bandwidth be $B_{r}$, the signal amplitude be $A_{a}$ and the carrier frequency of the radar system be $f_{c}$. The frequency range of the signal at this time is from $f_{c}-\frac{B_{r}}{2}$ to $f_{c}+\frac{B_{r}}{2}$. Thus, the wideband LFM signal can be expressed as

(1) $$S_{a}\left(t\right)=rect\left(\frac{t}{T_{r}}\right)A_{a}\exp\left(j\pi Kt^{2}+j2\pi f_{c}t\right)$$

where rect(·) is the rectangular function, specifically represented as follows:

(2) $$rect\left(\frac{t}{T}\right)=\left\{\begin{array}{cc} 1 & -\frac{T}{2}<t<\frac{T}{2}\\ 0 & \mathrm{otherwise} \end{array}\right.$$

To enhance the signal’s anti-jamming capability, the radar system generates a noise signal with the same time duration as the original signal. Given the same signal bandwidth, a random noise signal with a Gaussian power spectral density offers better range resolution compared to most other types of random noise signals, and its bandwidth is also easier to control. Therefore, a random noise signal with a Gaussian-shaped power spectral density was selected for this purpose. Let the Gaussian white noise signal be denoted as n(t). It is a zero-mean, wide-sense stationary random process, and its probability distribution is given by

(3) $$P\left(n\left(t\right)\right)=\frac{1}{\sqrt{2\pi }\sigma }\exp\left[-\frac{n^{2}\left(t\right)}{2\sigma^{2}}\right]$$

The signal has the following band-limited power spectrum:

(4) $$G\left(f\right)=\left\{\begin{array}{cc} \frac{\sigma^{2}}{\Delta F} & 0<f<\Delta F\\ 0 & \mathrm{otherwise} \end{array}\right.$$

where $\sigma^{2}$ represents the variance of the modulation noise.

(5) $$\sigma^{2}=2m_{fe}^{2}\Delta \Omega \int_{0}^{\Delta \Omega }\frac{1-\cos\Omega \tau }{\Omega^{2}}d\Omega$$

where ΔΩ=2πΔF denotes the bandwidth of the modulation noise and τ is the time delay. $m_{fe}=K_{FM}\sigma /\Delta F$ is the effective modulation index and $K_{FM}$ is the frequency modulation slope. Based on this noise signal, the radar system generates a baseband NFM signal with the following form:

(6) $$h\left(t\right)=A_{n}\exp\left[j2\pi K_{FM}\int_{0}^{t}n\left(t^{\prime }\right)dt^{\prime }\right]$$

where $A_{n}$ is the amplitude of the modulated signal, and the power spectrum of the NFM signal is then given by

(7) $$G_{h}\left(f\right)=A_{n}\int_{0}^{\infty }\cos\left(2\pi f\right)\tau \exp\left[-{m_{fe}}^{2}2\pi \Delta F\int_{0}^{\Delta F}\frac{1-\cos(2\pi f)\tau }{2\pi f^{2}}df\right]d\tau$$

When $m_{fe}\gg 1$, the power spectrum (7) of the NFM signal [14,15] can be approximated as

(8) $$G_{h}\left(f\right)=\frac{{A_{n}}^{2}}{2}\frac{1}{\sqrt{2\pi }K_{FM}\sigma }\exp\left[-\frac{f^{2}}{2{\left(K_{FM}\sigma \right)}^{2}}\right]$$

The power spectral density of the NFM signal $G_{h}\left(f\right)$ has a linear relationship with the probability density of the modulating noise P(n(t)). When the probability density of the random noise is Gaussian, the power spectral density of the NFM signal is also Gaussian. In this case, the half-power bandwidth of the NFM signal is $B_{j}=2\sqrt{2\ln2}K_{FM}\sigma$, which is independent of the modulation noise bandwidth ΔF and instead depends on the power of the modulating noise $\sigma^{2}$ and the frequency modulation slope $K_{F}M$. Therefore, controlling the power of the modulation noise can allow the effective bandwidth of the NFM signal to be adjusted. To reduce the sampling rate in the radar echo receiving system, the effective bandwidth of the NFM signal must be much smaller than the bandwidth of the LFM signal, i.e., $B_{j}\ll B_{r}$. Furthermore, the radar system uses the LFM signal as the carrier and modulates it with the NFM signal via amplitude modulation [17,18]. Thus, based on Equations (1) and (6), the transmitted signal γ(t) of the radar system can be expressed as

(9) $$\gamma \left(t\right)=h\left(t\right)S_{a}\left(t\right)=rect\left(\frac{t}{T_{r}}\right)A\exp\left[j2\pi f_{c}t+j\pi Kt^{2}+j2\pi \theta \left(t\right)\right]$$

where $\theta \left(t\right)=K_{FM}\int_{0}^{t}n\left(t^{\prime }\right)dt^{\prime }$ and $A=A_{a}A_{n}$ represents the amplitude of the transmitted signal. The instantaneous frequency of the transmitted signal from the radar system can be expressed as

(10) $$f\left(t\right)=f_{c}+Kt+K_{FM}n\left(t\right)$$

Since n(t) is Gaussian-distributed noise, its probability density function follows a zero-mean Gaussian distribution, satisfying $n\left(t\right)∼N\left(0,\sigma_{u}^{2}\right)$. Therefore, the probability density function of f(t) is a non-stationary process:

(11) $$f\left(t\right)∼N\left(f_{c}+Kt,K_{FM}^{2}\sigma_{u}^{2}\right)$$

From this, it can be observed that the instantaneous frequency of the transmitted signal in the radar system exhibits a certain degree of randomness, which helps maintain some level of anti-jamming capability during radar detection. However, this signal is still wideband, meaning that if the received target echo is processed directly, a high sampling rate would still be required. Therefore, upon receiving the target echo, the radar system can leverage the prior information about the transmitted signal generated by the system to perform relevant processing and extract the target information.

## 4. Signal Processing Method Based on de-chirp and Delay Matching

**Figure 3:**
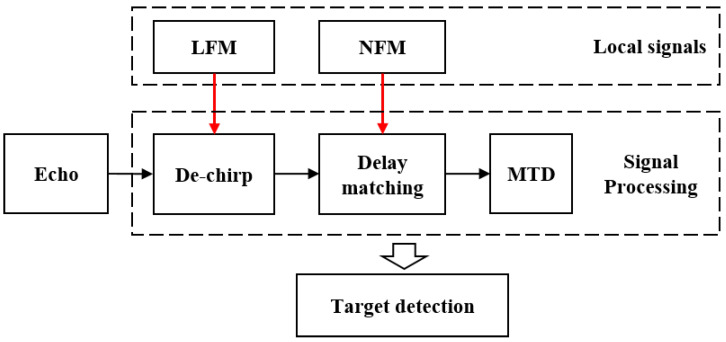
The structure of novel noise radar signal processing flow.

To efficiently process the detection echoes of the novel radar system’s composite waveform, a signal processing method based on de-chirp and delay matching is employed. The signal processing block diagram is shown in Figure 3. After performing de-chirp and delay matching with the radar system’s local signals [6,26], target detection is carried out using MTD.

**Figure 4:**
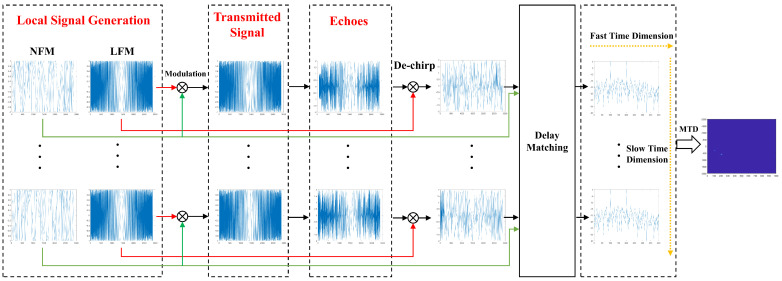
The visualized block diagram of novel noise radar signal processing (the specific delay matching structure will be introduced in the subsequent sections of the paper).

The visualized block diagram of the signal processing method is shown in Figure 4. In the fast time domain, our radar system first uses the transmitted LFM signal carrier as the reference signal to de-chirp the received echo, resulting in a narrowband NFM signal after removing the wideband LFM component. Consequently, subsequent signal processing requires a lower sampling rate. The system then performs delay matching between the transmitted NFM signal and the de-chirped echo, replacing the conventional matched filtering process. By analyzing the point frequency signal obtained from the matching process and considering the slope of the LFM signal, the time delay of the target echo is determined. Finally, in the slow time domain, MTD is conducted to acquire the target information.

Assuming that the target is at a distance *R* from our radar and moving at a velocity *v*, the corresponding target echo time delay is $\tau =\frac{2R}{c}$ and the Doppler frequency is $f_{d}=\frac{2vf_{c}}{c}$. Therefore, the target echo received by the radar system can be expressed as

(12) $$\gamma^{\prime }\left(t\right)=rect\left(\frac{t-\tau }{T_{r}}\right)A^{\prime }\exp\left[j2\pi \left(f_{c}+f_{d}\right)\left(t-\tau \right)+j\pi K{\left(t-\tau \right)}^{2}+j2\pi \theta \left(t-\tau \right)\right]$$

The de-chirp reference signal of our radar system is the transmitted LFM signal within the reception window, expressed as

(13) $$S_{d}\left(t\right)=rect\left(\frac{t-\tau }{T_{r}}\right)A_{a}\exp\left(j\pi Kt^{2}+j2\pi f_{c}t\right)$$

By conjugately multiplying the received target echo signal (12) with the de-chirp reference signal (13) in the radar system, the de-chirped signal can be obtained as

(14) $$\begin{array}{cc} \gamma_{d}\left(t\right) & =\gamma^{\prime }\left(t\right)\cdot conj\left[S_{d}\left(t\right)\right]\\ \; & =rect\left(\frac{t-\tau }{T_{r}}\right)A_{a}A^{\prime }\exp\left[j2\pi \left(f_{c}+f_{d}\right)\left(t-\tau \right)+j\pi K{\left(t-\tau \right)}^{2}+j2\pi \theta \left(t-\tau \right)\right]\\ \; & \;\cdot \exp(-j\pi Kt^{2}-j2\pi f_{c}t)\\ \; & =rect\left(\frac{t-\tau }{T_{r}}\right)A_{a}A^{\prime }\exp\left[j2\pi \left(f_{d}-K\tau \right)t+j2\pi \theta \left(t-\tau \right)+j\pi \left(K\tau^{2}-2f_{c}\tau -2f_{d}\tau \right)\right] \end{array}$$

where $K\tau^{2}-2f_{c}\tau -2f_{d}\tau$ can be considered as a constant, and thus it can be denoted as

(15) $$C=\exp[j\pi \left(K\tau^{2}-2f_{c}\tau -2f_{d}\tau \right)]$$

Therefore, the de-chirped signal in Equation (14) can be expressed as

(16) $$\gamma_{d}\left(t\right)=rect\left(\frac{t-\tau }{T_{r}}\right)A_{a}A^{\prime }C\exp\left[j2\pi \left(f_{d}-K\tau \right)t+j2\pi \theta \left(t-\tau \right)\right]$$

**Figure 5:**
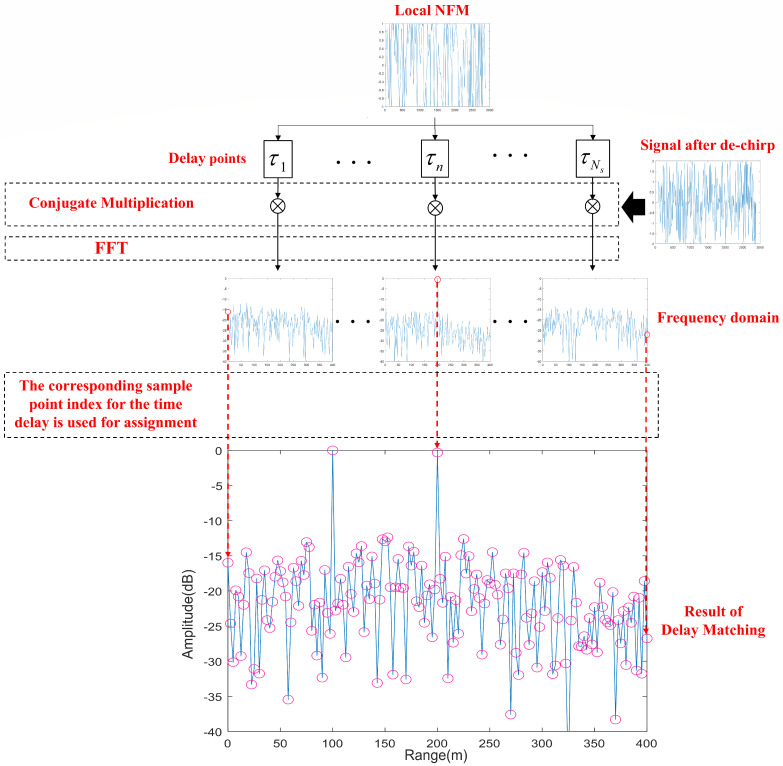
The visualized block diagram of delay matching (assuming that one processing period consists of $N_{s}$ sampling points, the time delay of a certain target is located at the *n*th sampling point).

After obtaining the de-chirped echo signal, the radar system performs delay matching to replace the conventional matched filtering process. The specific process flow is illustrated in Figure 5.

Based on the noise component in the radar system’s transmitted signal (6), the matched reference zero intermediate frequency NFM signal can be expressed as

(17) $$h_{0}\left(t\right)=A_{n}\exp\left[j2\pi \theta (t)\right]$$

The matching result can be obtained by multiplying the conjugate of the reference NFM signal $conj[h_{0}\left(t-\tau^{\prime }\right)]$ with different delays by the de-chirped echo signal $\gamma_{d}\left(t\right)$, which yields

(18) $$\begin{array}{cc} m(t) & =\gamma_{d}\left(t\right)\cdot conj\left[h_{0}\left(t-\tau^{\prime }\right)\right]\\ \; & =rect\left(\frac{t-\tau }{T_{r}}\right)A_{a}A^{\prime }A_{n}C\exp\left[j2\pi \left(f_{d}-K\tau \right)t+j2\pi \theta \left(t-\tau \right)-j2\pi \theta \left(t-\tau^{\prime }\right)\right] \end{array}$$

When the delay of the reference NFM signal during the delay matching process equals the target delay, i.e., $\tau^{\prime }=\tau$, the matching result (18) can be further expressed as

(19) $$m\left(t\right)=rect\left(\frac{t-\tau }{T_{r}}\right)A_{a}A^{\prime }A_{n}C\exp\left[j2\pi \left(f_{d}-K\tau \right)t\right]$$

It can be observed that, at this point, the matching result is a narrowband signal with a single frequency component at frequency $f_{d}-K\tau$. Based on the definitions of the variables and the typical magnitudes of commonly used parameters in radar systems, we take, for example, a target speed on the order of $10^{2}$, a target distance on the order of $10^{3}$, a carrier frequency on the order of $10^{9}$, a signal pulse width on the order of $10^{-6}$, and a signal bandwidth on the order of $10^{6}$. Therefore, we can derive

(20) $$\frac{f_{d}}{K\tau }=\frac{2vf_{c}cT_{r}}{c2RB_{r}}=\frac{vf_{c}T_{r}}{RB_{r}}\approx \frac{1}{10^{4}}\ll 1$$

Thus, Equation (19) can be approximated as

(21) $$m\left(t\right)=rect\left(\frac{t-\tau }{T_{r}}\right)A_{a}A^{\prime }A_{n}C\exp\left[j2\pi \left(-K\tau \right)t\right]$$

During the delay matching process, the result value corresponding to the delay sampling point index of each delay match is used as the value for the corresponding sampling point index in the overall matching result. Consequently, the matching result obtained after the entire delay matching process will exhibit a maximum peak at Kτ. Since the parameter *K* is known, we can convert this frequency domain matching result into an equivalent matched filtering result, thus obtaining the target’s delay τ and subsequently calculating the target’s distance. Additionally, as the Doppler component remains in the constant term within the frequency domain result, we can obtain the target’s velocity information to detect the target by further processing through slow time accumulation and MTD.

## 5. System Structure Based on de-chirp and Delay Matching

**Figure 6:**
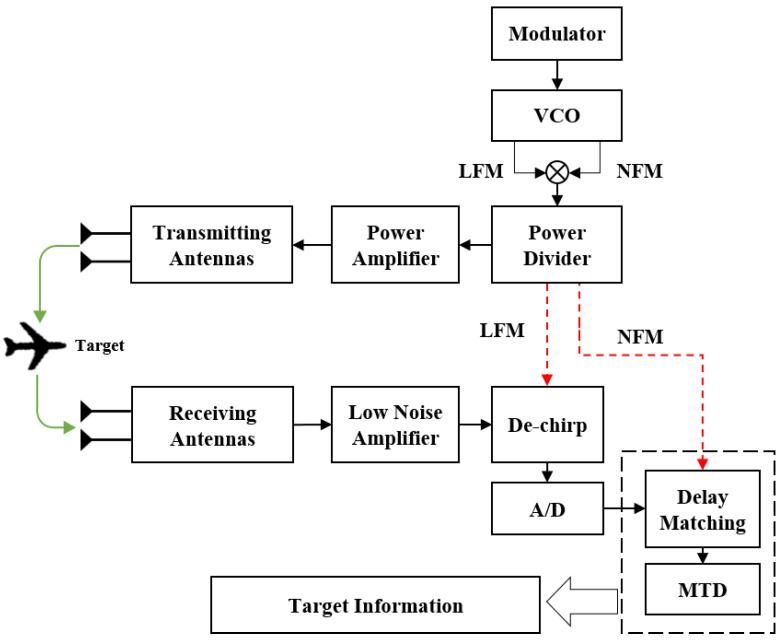
The structure of the improved radar system based on the proposed de-chirp and delay matching method.

Based on the conventional hardware architecture of the LFM continuous wave radar systems in Section 2, the system architecture proposed in this paper is illustrated in Figure 6. The main changes are found in the local signal generation part at the transmitter and the echo signal processing part at the receiver.

During operation, the local signal generation module simultaneously produces both a wideband LFM signal and a narrowband NFM signal. These two signals are combined through modulation to form the novel composite signal transmitted by the radar system, while portions of the generated signals are reserved for processing the received echoes. Upon receiving echoes scattered by a moving target, the radar’s receiving module performs de-chirping using the reserved LFM signal, yielding a narrowband NFM signal with a frequency difference. At this point, the narrowband signal is digitized using A/D conversion at a lower sampling rate. The reserved NFM signal is then used for delay matching to obtain results similar to matched filtering. Finally, target detection is achieved through MTD by accumulating results over multiple slow time periods. This system architecture generates complex transmitted waveforms that are resistant to interference and difficult to recognize. Additionally, by reducing the sampling rate on the receiving end, the system significantly reduces implementation costs.

## 6. Simulation Experimental Results

We simulated the radar system’s target detection process, with the simulation parameters listed in Table 1. The bandwidth of the LFM signal is set based on commonly used radar operational scenarios. To significantly reduce the subsequent sampling rate, we set the bandwidth of the NFM signal by reducing the LFM signal’s bandwidth by one order of magnitude. The simulation generated LFM and NFM signals of the same time width. The LFM signal was a wideband signal, while the NFM signal was a narrowband signal. During the signal generation phase, a high sampling rate was used to process the wideband signal, while in the signal processing phase only a lower sampling rate was needed for the narrowband NFM signal. Two moving targets with different distances and velocities were set in the simulation to validate the detection performance of the radar system under the proposed signal processing method.

**Figure 7:**
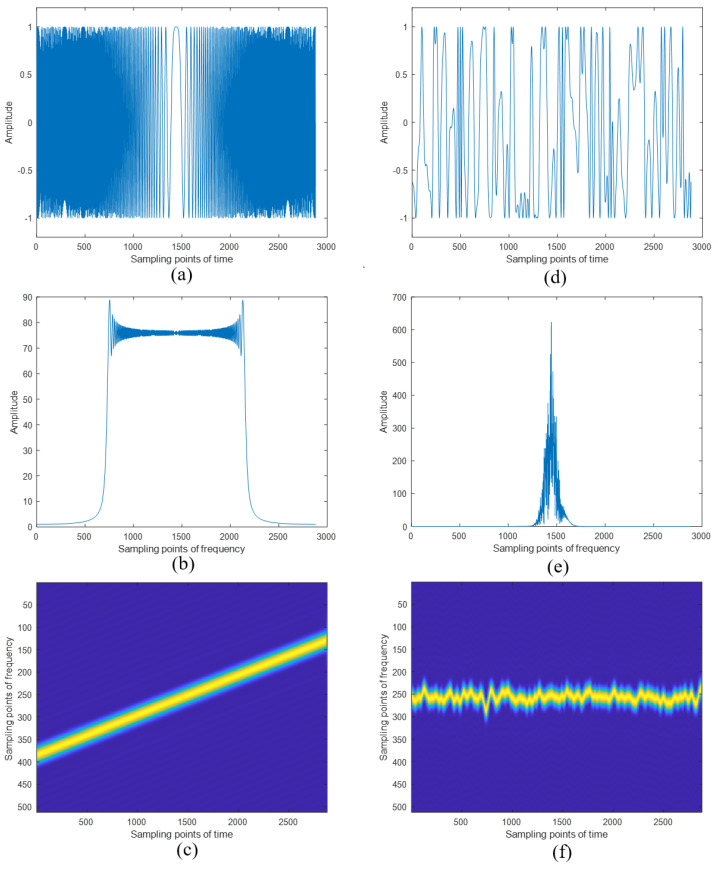
The LFM and NFM signals generated locally by the radar system. (**a**) The time domain waveform of LFM; (**b**) the frequency domain waveform of LFM; (**c**) the time–frequency domain waveform of LFM; (**d**) the time domain waveform of NFM; (**e**) the frequency domain waveform of NFM; (**f**) the time–frequency domain waveform of NFM.

First, we simulated the LFM signal to be modulated and the NFM signal based on the set simulation parameters and the generation principle of the novel designed radar signal. A wideband LFM signal and a narrowband NFM signal were generated. The time domain, frequency domain, and time–frequency domain waveforms of the signals are shown in Figure 7. Under the same signal time width, the wideband LFM signal exhibited a broad frequency range, while the narrowband NFM signal exhibited the characteristics of noise with a random frequency distribution. On the other hand, it is evident that the characteristics of the LFM signal can be easily analyzed. In contrast, due to its strong randomness, it is challenging to extract the characteristic parameters of the NFM signal.

**Figure 8:**
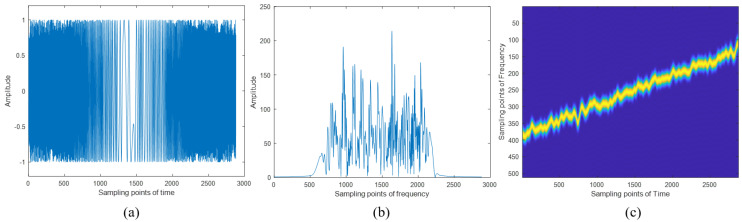
The transmitted signal of the radar system. (**a**) The time domain waveform of the transmitted signal; (**b**) the frequency domain waveform of the transmitted signal; (**c**) the time–frequency domain waveform of the transmitted signal.

The LFM signal was used as the carrier signal, and modulation was performed using the NFM signal. The composite signal waveform transmitted by our radar system is shown in Figure 8. The transmitted signal exhibits strong randomness both in the time domain and frequency domain, making it difficult to recognize. In the frequency domain, the transmitted signal appears as a wideband signal, which still requires a high sampling rate.

**Figure 9:**
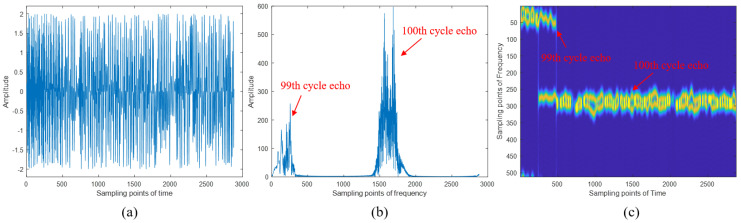
The echo signal after de-chirp. (**a**) The time domain waveform of the de-chirped signal; (**b**) the frequency domain waveform of the de-chirped signal; (**c**) the time–frequency domain waveform of the de-chirped signal.

After the generation of the transmitted signal, delay information and Doppler information were added to the echoes according to the set distances and speeds of the two targets. Then, by multiplying the conjugate of the locally generated LFM signal, the de-chirped echo signal was obtained. Taking the echo signal from the 100th cycle as an example, the waveform of the de-chirped echo signal within a single period is shown in Figure 9. Based on the comparison between Figure 8b and Figure 9b, we can clearly observe the difference in spectral width of the echo signals before and after the de-chirping process. The de-chirping significantly reduces the spectral bandwidth of the echo signals. The de-chirped signal retains only the NFM signal component, resulting in a narrowband signal, which allows for subsequent processing with a lower sampling rate. Due to the nature of continuous wave radar echo processing, where a portion of the transmitted signal from the previous cycle may still be present within the processing window of the current cycle, Figure 9 includes part of the signal from the 99th cycle.

**Figure 10:**
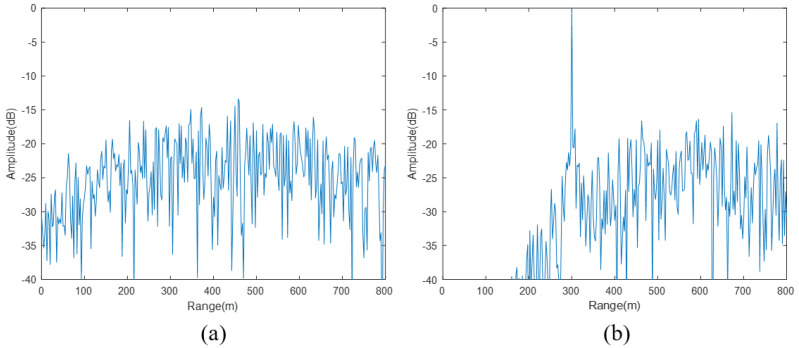
The comparison chart of the delay-matching traversal process. (**a**) Frequency domain result of the product when the delay does not correspond to a target; (**b**) frequency domain result of the product when the delay corresponds to one target.

The locally generated NFM signal is conjugately multiplied with the de-chirped signal after applying different time delays, and this process is repeated for all sampling points within a processing cycle. The results obtained at each step of the delay matching process are shown in Figure 10. When the applied delay does not correspond to any of the target delays, no peak appears in the frequency domain of the product result. However, when the delay matches one of the target delays, a peak appears at the corresponding sampling point of the target delay in the frequency domain of the product result.

**Figure 11:**
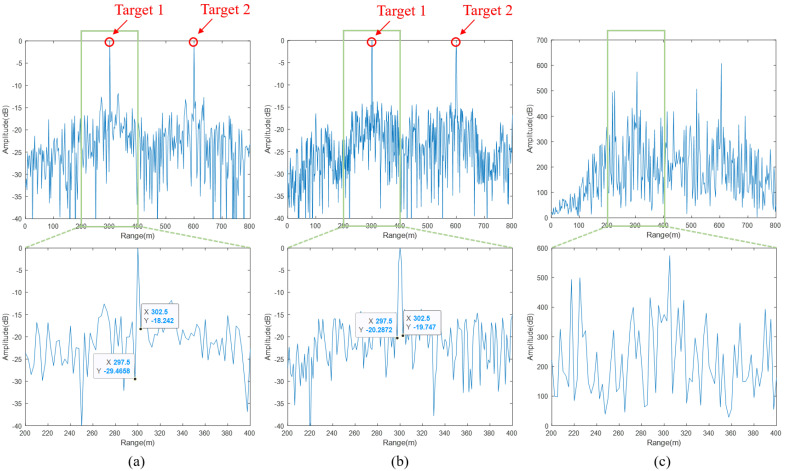
Processing results for a single cycle in the fast time dimension. (**a**) The result of delay matching; (**b**) the result of matched filtering; (**c**) the result of traditional de-chirp and frequency domain processing.

For a single cycle, the value corresponding to the delay index sampling point in the frequency domain result of each traversal is taken as the value of the corresponding sampling point in the matching result for that cycle. The fast time processing results of the 100th cycle’s echo are shown in Figure 11. Figure 11a shows the range domain result obtained by delay-matching between the local NFM signal and the de-chirped echo. Figure 11b presents the range domain detection result obtained by the proposed composite transmitted signal directly matched with the echo signal. The results of the traditional method, which uses de-chirp and frequency domain processing as a substitute for matched filtering, are shown in Figure 11c. Evidently, this approach is not suitable for the radar system with the signal structure proposed in this paper, as it fails to detect the target. Both the delay matching result in Figure 11a and the matched filtering result in Figure 11b successfully obtained the range information of the two targets. According to the bandwidth of the local LFM signal set in the simulation parameters, the range resolution of the original signal can be calculated as 5 m. In our approach, using de-chirping delay-matching processing, although the required sampling rate is reduced by lowering the spectral bandwidth, the results shown in Figure 11a,b indicate that the main lobe width of the range domain matching result still maintains a range resolution level of 5 m. Therefore, the proposed processing method does not affect the system’s range resolution. Moreover, the main-to-side lobe ratio in the delay matching method’s result was −22.63 dB, while that of the matched filtering result was −22.93 dB, showing only a minor difference. Thus, the proposed method retains the range resolution capability of the original signal and maintains a similar main-to-side lobe ratio even with the reduced sampling rate. In the simulation, the traditional matched filtering method requires a sampling rate of 120 MHz, whereas the proposed method achieves the same performance with only a 60 MHz sampling rate.

**Figure 12:**
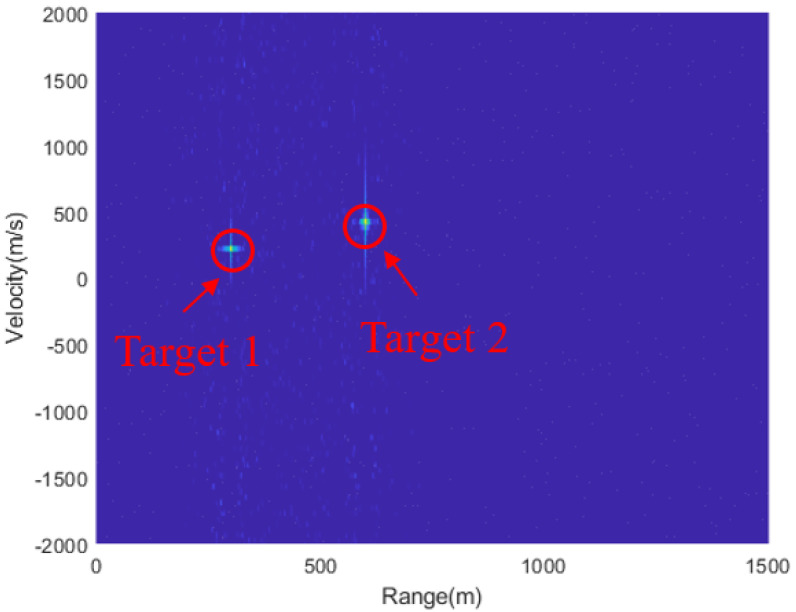
The range–Doppler map result of the de-chirp and delay matching method.

After obtaining the delay-matching result, MTD accumulation is performed over multiple processing cycles in the slow time dimension to achieve the targets’ range–Doppler map, as shown in Figure 12. It can be observed that both targets are successfully detected.

## 7. Conclusions

This paper addresses the limitations of traditional radar systems, specifically the use of simple transmission signals and the high sampling rate requirements, by introducing a novel composite transmission waveform that combines narrowband NFM and LFM signals. The proposed signal optimization method, based on de-chirp processing and delay matching, offers a promising approach to processing this complex waveform. The method effectively reduces the radar system’s sampling rate requirements without compromising key performance metrics such as sidelobe levels and range resolution. These metrics remain comparable to those achieved with direct processing at higher sampling rates. Simulation experiments were conducted to verify the validity and complexity of the composite signal. The de-chirp processing successfully isolates the narrowband difference-frequency NFM signal, where the resulting frequency differences reflect the echo delay information. By applying delay matching in each processing cycle, the proposed method achieves results akin to traditional matched filtering techniques, ensuring accurate detection and range estimation. Moreover, through slow time accumulation, additional target information is extracted, enhancing the system’s detection capabilities. However, a notable drawback of the delay matching method is the computational burden, as it requires a number of delay matching operations proportional to the total number of sampling points in each processing cycle. This increased computational load may affect real-time processing performance in more complex scenarios. Finally, although the proposed method demonstrates promising results in terms of reducing sampling rate requirements and maintaining detection performance, future work should focus on improving the system’s robustness against strong direct wave interference and environmental clutter. Further studies could explore optimization strategies for minimizing computational complexity while enhancing interference suppression capabilities.

## Figures and Tables

**Table 1:** The simulation parameters.

Parameters	Values
LFM bandwidth	60 MHz
LFM timewidth	24 μs
NFM bandwidth	4 MHz
NFM timewidth	24 μs
Carrier frequency	600 MHz
Processing repetition interval	24 μs
Repetition numbers	256
Signal generation sampling rate	120 MHz
Signal processing sampling rate	60 MHz
Target ranges	300 m, 600 m
Target speeds	200 m/s, 400 m/s

## Data Availability

Data are contained within the article.
